# Preclinical immunogenicity and safety evaluation of MDCK cell-derived quadrivalent influenza virus subunit vaccine

**DOI:** 10.3389/fimmu.2026.1827059

**Published:** 2026-06-26

**Authors:** Shuang Li, Jialin Lv, Xuejiao Li, Yue Yang, Yimeng Yan, Lina Sui, Yehong Wu

**Affiliations:** 1 Changchun Institute of Biological Products Co., Ltd, Changchun, China; 2 State Key Laboratory of Novel Vaccines for Emerging Infectious Diseases, China National Biotec Group Company Limited, Beijing, China

**Keywords:** influenza vaccine, MDCK cell-derived, quadrivalent subunit vaccine, vaccine immunogenicity, vaccine safety

## Abstract

To address the limitations of egg-based influenza vaccine production, serum-free suspension culture of Madin-Darby Canine Kidney cells (MDCK) has emerged as a promising alternative. In this study, a quadrivalent influenza virus subunit vaccine was developed using this platform by culturing four viral strains, followed by purification, inactivation, and formulation. Preclinical safety evaluations in rats, rabbits, and guinea pigs demonstrated no systemic toxicity. In mice, immunization with the vaccine elicited significant hemagglutination inhibition and microneutralization antibodies against H1N1, H3N2, BY, and BV strains at 28 and 42 days post-vaccination. These results indicate that the vaccine candidate possesses a favorable safety profile and induces robust immunogenicity.

## 1 Introduction

Influenza is a severe viral respiratory disease, with annual global pandemics characterized by high morbidity and mortality rates (1, 2). It poses a serious threat to public health and imposes a significant economic burden worldwide, representing a major public health challenge (3). Although egg-based influenza vaccines, available since the 1940s, have a mature production process and remain dominant in the market, they present several inherent drawbacks (4). These include dependence on large quantities of chicken eggs, which often constrains the production capacity once the World Health Organization (WHO) announces the annual strain composition (5, 6). The process of inoculating individual eggs leads to batch-to-batch variability in yield and quality, while the use of standard eggs elevates the risk of adventitious agent contamination, complicating quality control (7, 8). Furthermore, serial virus passage in eggs can induce antigenic changes, potentially reducing vaccine effectiveness due to mismatch with circulating strains. Although residual egg protein content has been reduced in modern vaccines, concerns regarding egg-related allergies persist (9). An outbreak of avian influenza could also disrupt egg supply, further limiting vaccine availability (10).

To overcome these limitations, the use of animal cell lines for human influenza vaccine production has gained increasing attention in recent years (11). As early as 1995, the WHO recommended mammalian cell culture as a substrate to enhance production efficiency and vaccine protection (12). Cell-based production offers multiple advantages: it simplifies operations, facilitates scale-up in bioreactors, shortens production cycles, and increases yield (13). During a pandemic, it ensures a flexible and timely response independent of egg supply. The use of defined culture materials reduces allergenic risks, and viruses propagated in cells are less prone to antigenic drift, potentially improving the match to circulating strains and vaccine efficacy. Additionally, cell banks are thoroughly characterized and tested, further minimizing the risk of adventitious agent contamination.

Common mammalian cell lines used for influenza virus cultivation include MDCK, African green monkey kidney epithelial (Vero), and human embryonic retina cells immortalized with adenovirus type 5 E1 genes (PER.C6) cells. Among these, MDCK cells demonstrate higher susceptibility to infection and can yield up to an order of magnitude virus than Vero cells, making them the preferred substrate for many vaccine developers (14). Upstream processes for influenza virus growth in MDCK cells can be adherent or suspension-based. Adherent culture is relatively cumbersome, often requires trypsin and serum, carries a higher contamination risk, and is less scalable. “In contrast, serum−free suspension culture simplifies processing by eliminating the need for serum and trypsin. This approach not only reduces contamination risks but also facilitates seamless scale−up to meet pandemic demand, establishing it as the preferred method for cell−based influenza vaccine development (15, 16).

Currently approved cell-based influenza vaccines primarily utilize MDCK or Vero cell substrates (17). For instance, in 2001, Solvay’s trivalent subunit vaccine (Influvac), produced using serum-free microcarrier-based adherent MDCK culture, was approved in the Netherlands (18), though it was not formally launched due to supply delays. Subunit vaccines based on suspension MDCK cells from Seqirus (Australia) and SK Chemicals (South Korea) have since gained approval in Europe, Australia, the United States, and South Korea. Notably, in late 2020, the United States Food and Drug Administration (U.S. FDA) approved Seqirus’s MF59-adjuvanted H5N1 pandemic subunit vaccine produced using serum-free suspension MDCK technology (19). Both companies employ serum-free suspension MDCK platforms, highlighting the industrial adoption of this advanced production approach. During the seasons evaluated, cell-based quadrivalent inactivated influenza vaccines was approved for people aged ≥4 years and has been approved for ≥6 months since 2021 (20). Previous real world observational studies have shown that cell-based quadrivalent inactivated influenza vaccine is more effective at preventing influenza-associated outcomes compared to egg-based quadrivalent inactivated influenza vaccine (13).

## 2 Results

### 2.1 Safety evaluation

#### 2.1.1 No abnormalities were observed in the single-dose toxicity study of intramuscular injection in SD rats

**Figure 1:**
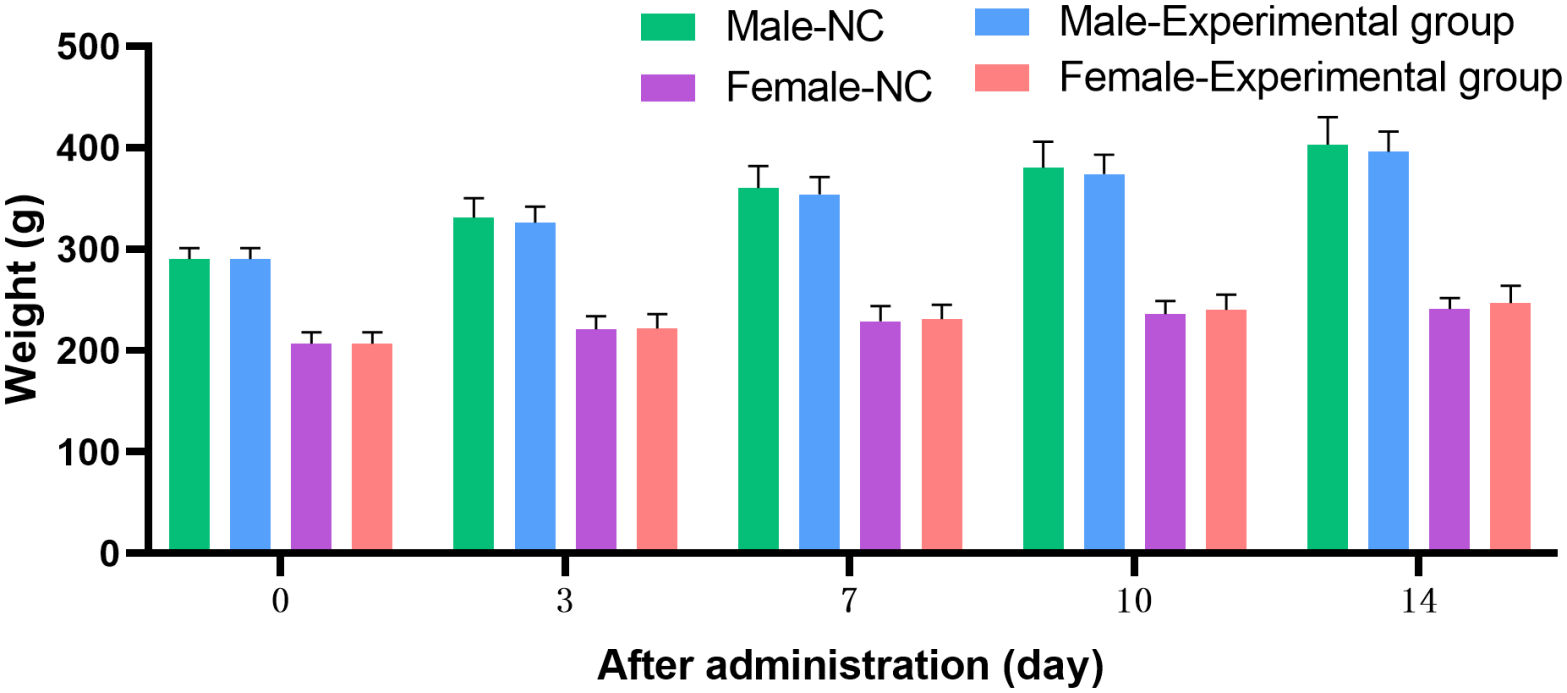
Effect of single intramuscular administration on body weight in SD rats. Animals received an intramuscular injection of 1.0 mL/rat. The negative control group was administered 0.9% sodium chloride injection, while the test group received the quadrivalent influenza virus subunit vaccine (MDCK cell-derived). Data points represent the mean value for 20 rats per group.

A single-dose toxicity study was conducted in SD rats via intramuscular injection to evaluate potential direct damage and establish a clinical safety range. Administration of the quadrivalent influenza virus subunit vaccine (MDCK cell-derived) resulted in no abnormal clinical or local symptoms, and no mortality was observed throughout the study period. Animals exhibited normal body weight gain (**Figure 1**). Necropsy of surviving animals at the end of the recovery period revealed no significant gross pathological changes in tissues or organs. Histopathological examination was not performed.

#### 2.1.2 No abnormalities were observed in the repeated-dose toxicity study in SD rats by intramuscular injection

**Figure 2:**
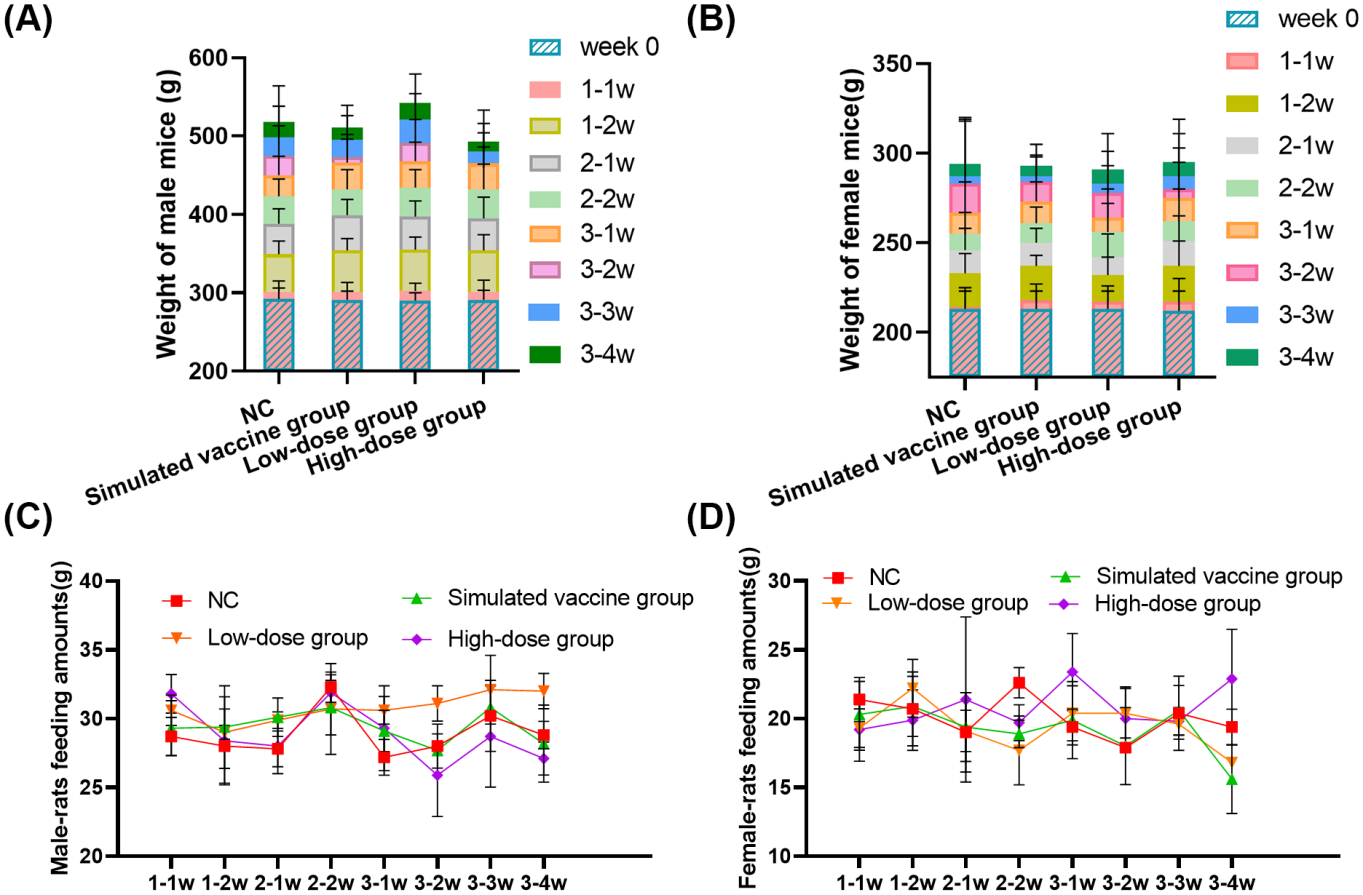
Effects of repeated intramuscular administration on body weight and food intake in SD rats. The negative control group received 0.9% sodium chloride injection, and the mock vaccine control group was given formulation buffer, both at a volume of 0.5 mL/rat. The low-dose and high-dose groups were administered the quadrivalent influenza virus subunit vaccine (MDCK cell-derived) at volumes of 0.25 mL/rat and 0.5 mL/rat, respectively. Clinical signs were monitored daily throughout the study, while body weight **(A, B)** and food consumption **(C, D)** were measured weekly. Here, “1-1w” refers to the first week after the initial injection, and so forth. Each data point represents the mean value from 20 rats.

To evaluate the safety of the vaccine and simulated vaccines, predict possible adverse reactions when used in large-scale populations, and reduce the risks borne by clinical trial subjects and clinical users, we administered quadrivalent influenza virus subunit vaccine (MDCK cell-derived) to SD rats multiple intramuscular injections to observe their toxic reactions and their severity, including the impact on immune organs and virulent target organs, and the reversibility of toxicity. During the study period, no significant abnormal clinical or local signs were observed in any dose group. There were no animal deaths or moribund states. Animals in the simulated vaccine control, low-dose, and high-dose groups showed normal weight gain (**Figures 2A, B**) and no significant abnormalities in food consumption (**Figures 2C, D**). No significant abnormalities in Ophthalmic examination and urinalysis were found in any group. No significant abnormalities in serum biochemistry, hematology and coagulation were noted.

##### 2.1.2.1 CD4^+^/CD8a^+^ T-cell detection

**Figure 3:**
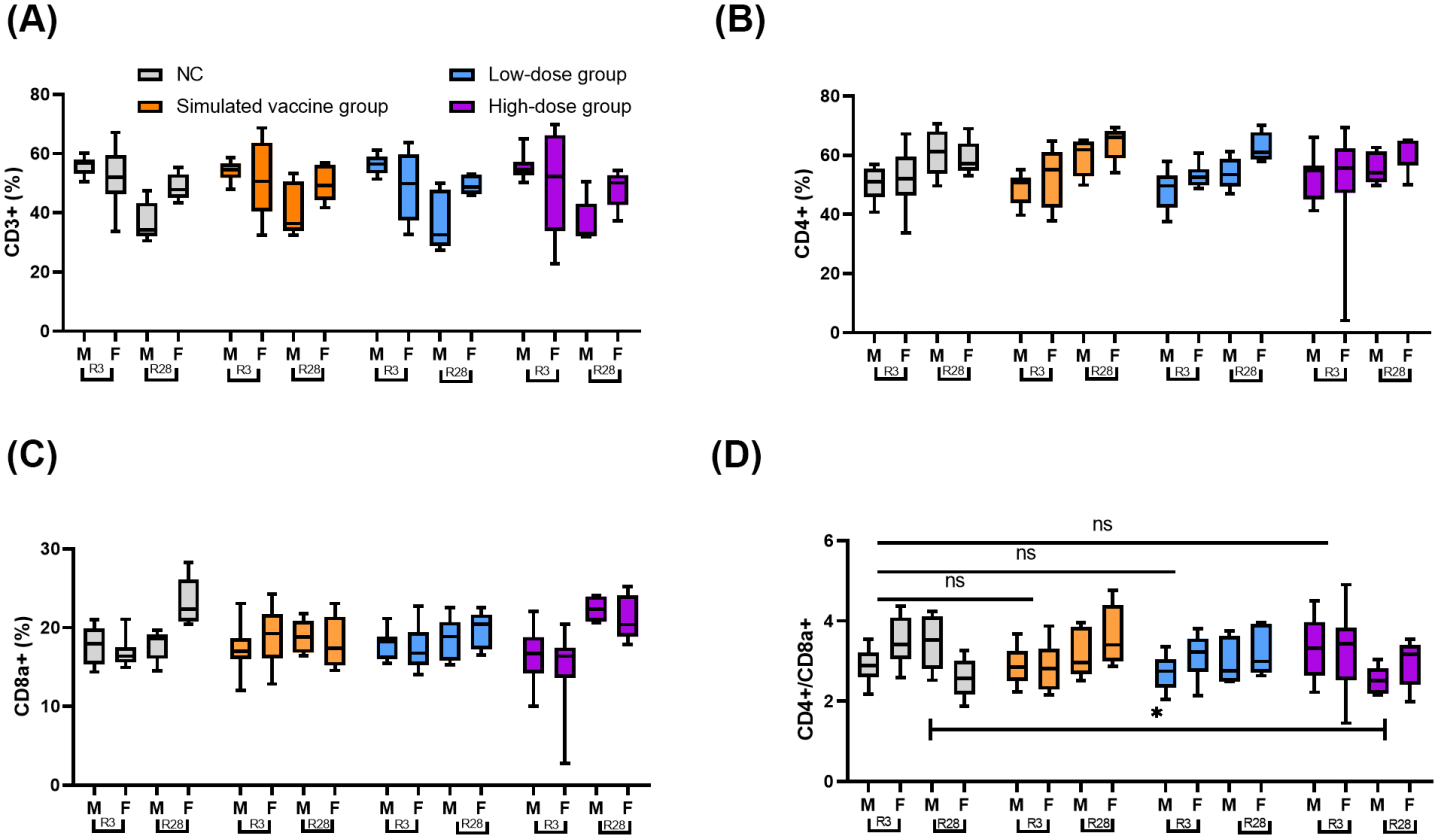
Effects of repeated intramuscular administration on CD4^+^ and CD8a^+^ T-cell counts in SD rats. During detection, a certain number of cells were collected using flow cytometry, and the counts of CD3^+^ **(A)**, CD4^+^ **(B)** and CD8a^+^ **(C)** cells were measured. The proportions of CD4^+^ cells and CD8a^+^ cells **(D)** among lymphocytes were analyzed using GuavaSoft software, and their ratios were calculated. Box-and-whisker plots represent group medians (middle line), 25th and 75th percentiles (box), and min and max (lower and upper whiskers). Statistical comparisons were performed using ANOVA and Tukey’s multiple comparison test; *p ≤ 0.05; ns, no significant difference.

To evaluate the immune function and assess the safety of the vaccine and the mock vaccine, we quantified CD3^+^CD4^+^ and CD3^+^CD8a^+^ cell populations and analyzed their proportions among lymphocytes, as well as the CD4^+^/CD8^+^ ratio. Results indicated that 3 days after the final administration, in the negative control group, the percentages of CD3^+^ and the CD4^+^/CD8a^+^ ratio were (55.97 ± 3.19)% (M) and (51.79 ± 10.51)% (F), (2.88 ± 0.40) (M) and (3.47 ± 0.59) (F), respectively. In the mock vaccine group, the corresponding values were (54.06 ± 3.29)% (M) and (51.19 ± 12.35)% (F) for CD3^+^, (2.88 ± 0.48) (M) and (2.85 ± 0.57) (F) for the CD4^+^/CD8a^+^ ratio. In the low-dose group, the results were (56.25 ± 3.43)% (M) and (49.63 ± 11.32)% (F) for CD3^+^, (2.71 ± 0.42) (M) and (3.12 ± 0.55) (F) for the CD4^+^/CD8a^+^ratio. In the high-dose group, the values were (55.25 ± 4.09)% (M) and (48.84 ± 16.23)% (F) for CD3^+^, (3.33 ± 0.78) (M) and (3.3 ± 0.98) (F) for the CD4^+^/CD8a^+^ ratio. No statistically significant differences were observed relative to the negative control group (**Figure 3**).

On day 28 after the last administration (R28), the number of CD8a^+^ cells in male animals of the high−dose group was (22.36 ± 1.61)%, which showed a statistically significant increase compared with that in the negative control group (17.82 ± 2.00)%. As this finding was isolated, lacked temporal continuity, and was not accompanied by abnormalities in other safety parameters, it was deemed unrelated to the test article.

##### 2.1.2.2 Cytokine detection

**Figure 4:**
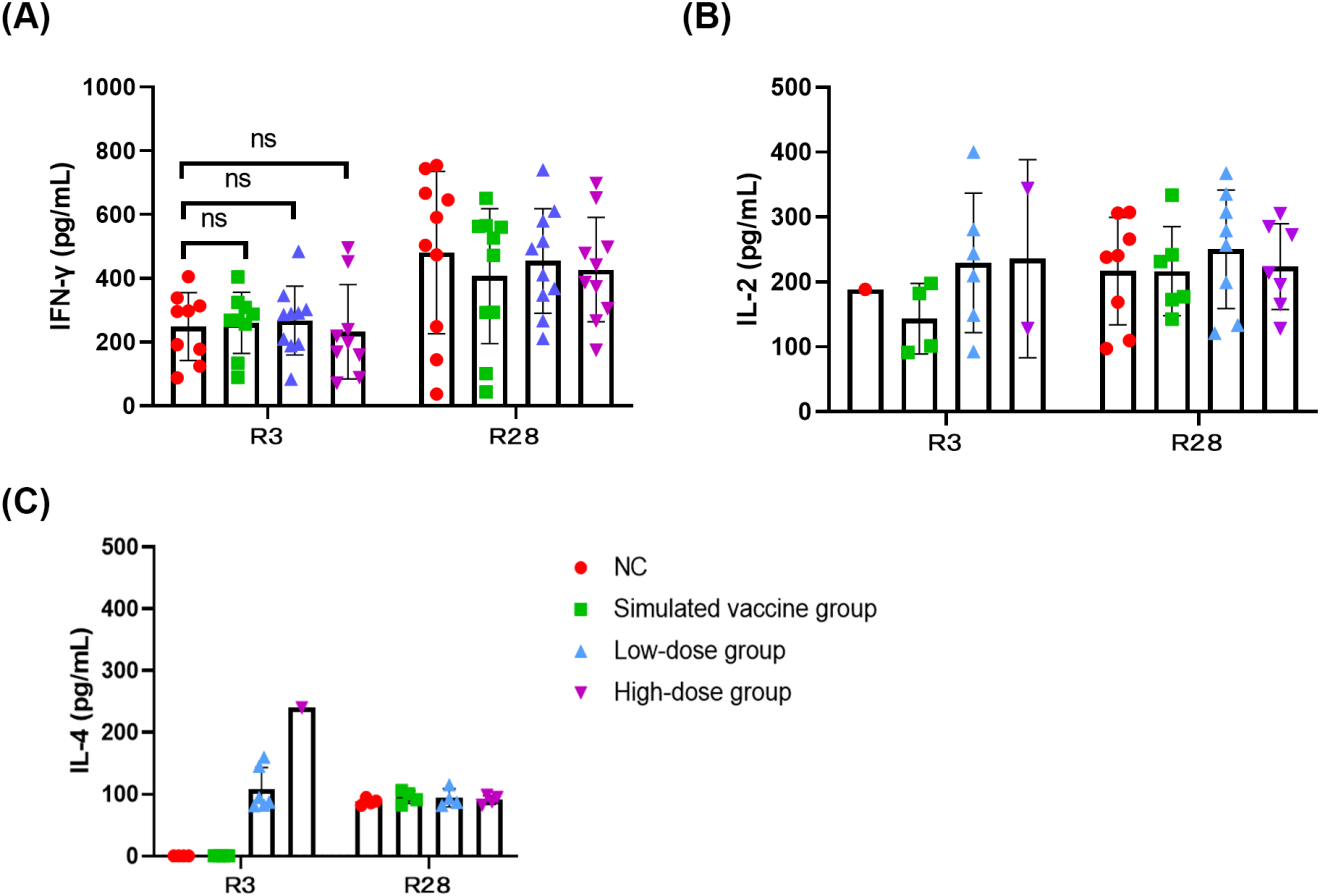
Effects of repeated intramuscular administration on cytokine levels in SD rats. Cytokines IFN-γ **(A)**, IL-2 **(B)**, and IL-4 **(C)** were measured by cytometric bead array. Each data point represents an individual animal. Statistical analysis was performed using ANOVA followed by Tukey’s multiple comparison test; ns, no significant difference.

Three days after the last administration (R3), interferon-γ (IFN-γ) levels in male animals across all dose groups fell below the lower limit of quantification (LLOQ). In females, IFN-γ was (260.24 ± 95.93) pg/mL, no significant differences were observed compared with the negative control (248.38 ± 106.69) pg/mL. At R28, no significant differences were found in either sex (**Figure 4A**).

At R3, elevated interleukin-2 (IL-2) levels were noted in individual male animals in the vaccine control (91.54 pg/mL) and low-dose groups (92.55 pg/mL), while the remaining animals showed IL-2 levels below the quantification limit. In female animals, the vaccine control was (160.57 ± 51.66) pg/mL, low-dose groups was (256.84± 93.77) pg/mL and high-dose groups was (470.71± 420.83) pg/mL, IL-2 levels did not differ significantly from those in the negative control group (188.42 pg/mL). By R28, IL-2 levels in all treatment groups showed no significant deviations from the negative control (**Figure 4B**).

At R3, increased interleukin-4 (IL-4) levels were detected in individual animals in the low-dose groups (113.79± 36.13 pg/mL for female, 82.89 pg/mL for male) and high-dose groups (239.69 pg/mL); all other animals had IL-4 levels below the quantification limit. At R28, IL-4 levels in the treatment groups were largely comparable to those in the negative control group. In the negative control groups, interleukin-4 (IL-2) levels were (88.38 ± 8.94) pg/mL (M) and (87.35 ± 2.01) pg/mL (F); in the mock vaccine groups were (99.3 ± 7.58) pg/mL (M) and 82.71 pg/mL (F); in the low-dose groups were (84.2 ± 3.49) pg/mL (M) and (104.14 ± 15.41) pg/mL (F) and in the high-dose were (93.59 ± 5.32) pg/mL (M) and 82.42 pg/mL (F) (**Figure 4C**).

No significant abnormalities were detected in anti-nuclear antibody levels by indirect immunofluorescence performed on serum aliquots obtained from biochemistry samples. Since hematological parameters in bone marrow exhibited no changes associated with the test article, microscopic examination was not conducted.

Pathological examination of all groups revealed that organ wet weights and their coefficients showed no significant abnormalities at R3 and R28. Furthermore, no test article- or formulation buffer-related histopathological changes were observed in any tissues or organs at either R3 or R28.

#### 2.1.3 Muscle irritation test in rabbits

In a safety study evaluating local irritation and its reversibility at the administration site following a single intramuscular injection of a quadrivalent influenza virus subunit vaccine (MDCK cell-derived) in Japanese large-eared white rabbits, gross observation 48 hours after injection revealed no local reactions (redness, swelling, congestion, exudation, degeneration, or necrosis) at either the administration or control sites in any animal. Observations after an additional 14-day recovery period likewise showed no such local reactions.

**Figure 5:**
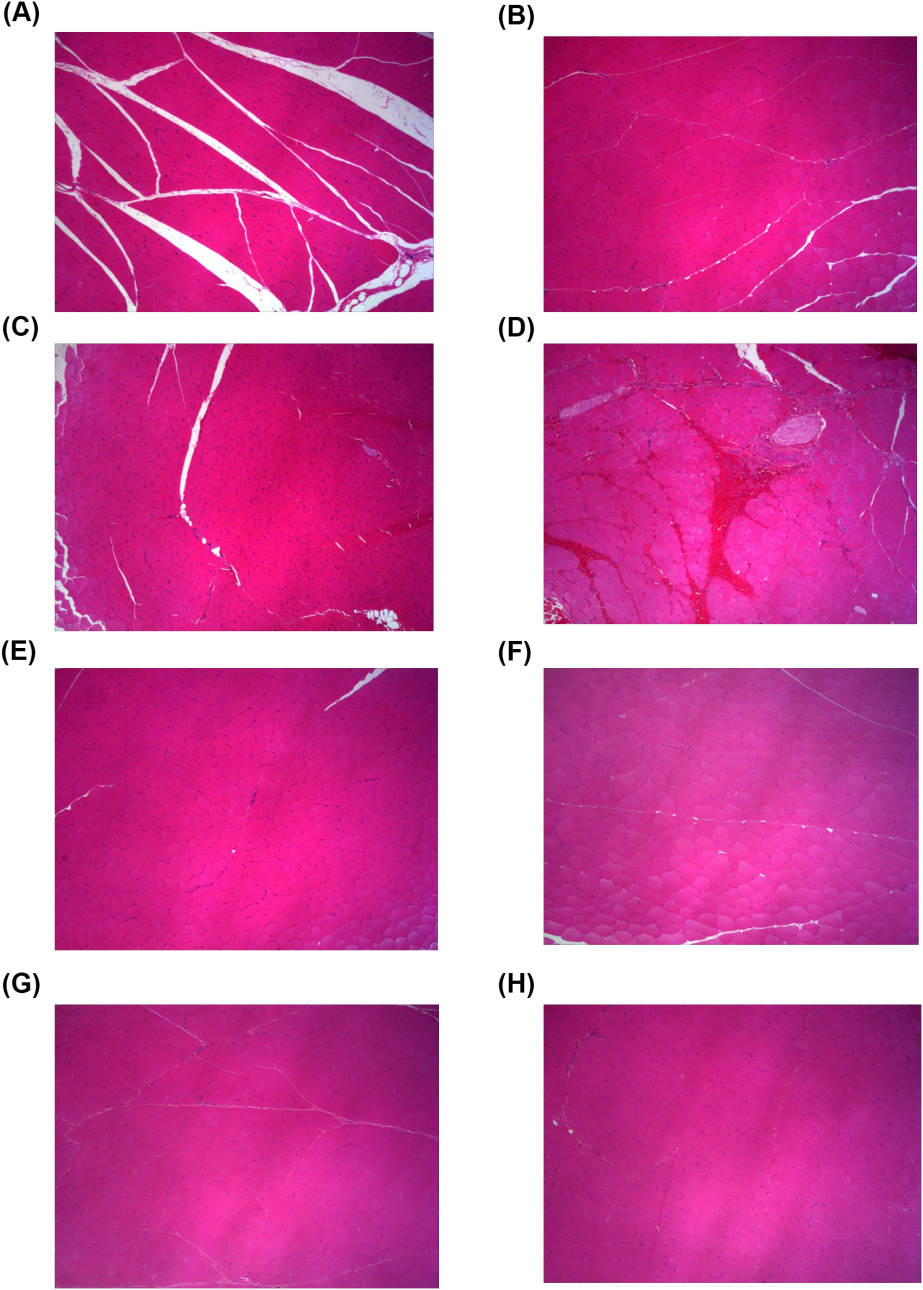
Rabbit muscle irritation test. Intramuscular injection was performed using a self-controlled design in the same animal. **(A)** Left quadriceps (control side), 48 h after administration, 40×; **(B)** Right quadriceps (control side), 48 h after administration, 40×; **(C)** Left quadriceps (control side), 48 h after administration, 40×; **(D)** Right quadriceps (control side), 48 h after administration, 40×; **(E)** Left quadriceps (control side), end of recovery period, 40×; **(F)** Right quadriceps (control side), end of recovery period, 40×; **(G)** Left quadriceps (control side), end of recovery period, 40×; **(H)** Right quadriceps (control side), end of recovery period, 40×.

Histopathological assessment further indicated that no test article-related histopathological changes were observed at either site 48 hours after dosing or following the recovery period (**Figure 5**).

#### 2.1.4 Active systemic anaphylaxis test in guinea pigs

The results showed that animals in the negative control group exhibited no significant allergic symptoms within 30 minutes after both challenge doses. In contrast, positive control animals displayed immediate symptoms (including restlessness, nose scratching, coughing, urination, defecation, dyspnea, unsteady gait, and convulsions) after the first administration (**Table 1**) and died within 5 minutes. Similar symptoms and outcomes were observed after the second administration (**Table 2**), confirming a strongly positive anaphylactic response. Animals in the low- and high-dose test article groups showed no significant allergic symptoms within 30 minutes after either challenge, indicating a negative anaphylactic reaction.

**Table 1:** List of guinea pig symptoms(first administration).

Group	Negative control group	Positive control group	Test article low dose group	Test article high dose group
Anxiety	0/4	4/4	0/4	0/4
Nose scratching	0/4	4/4	0/4	0/4
Coughing	0/4	4/4	0/4	0/4
Urination Spasm	0/4	3/4	0/4	0/4
Defecation	0/4	3/4	0/4	0/4
Difficulty breathing	0/4	4/4	0/4	0/4
Unsteady gait	0/4	4/4	0/4	0/4
Spasm	0/4	4/4	0/4	0/4
Death	0/4	4/4	0/4	0/4

**Table 2:** List of guinea pig symptoms(second administration).

Group	Negative control group	Positive control group	Test article low dose group	Test article high dose group
Anxiety	0/4	4/4	0/4	0/4
Nose scratching	0/4	4/4	0/4	0/4
Coughing	0/4	4/4	0/4	0/4
Urination Spasm	0/4	2/4	0/4	0/4
Defecation	0/4	3/4	0/4	0/4
Difficulty breathing	0/4	4/4	0/4	0/4
Unsteady gait	0/4	4/4	0/4	0/4
Spasm	0/4	4/4	0/4	0/4
Death	0/4	4/4	0/4	0/4

#### 2.1.5 Fertility and early embryonic development, and embryo-fetal development toxicity in SD rats by intramuscular injection

The effects of the quadrivalent influenza virus subunit vaccine (MDCK cell-derived) on feed intake, body weight, fertility, and embryo-fetal development were evaluated following repeated intramuscular administration in SD rats, in order to provide references for clinical safety. Results indicated that no significant abnormalities were observed in clinical observations for male or female rats across all groups. Similarly, no significant abnormalities were noted in food consumption or body weight.

**Figure 6:**
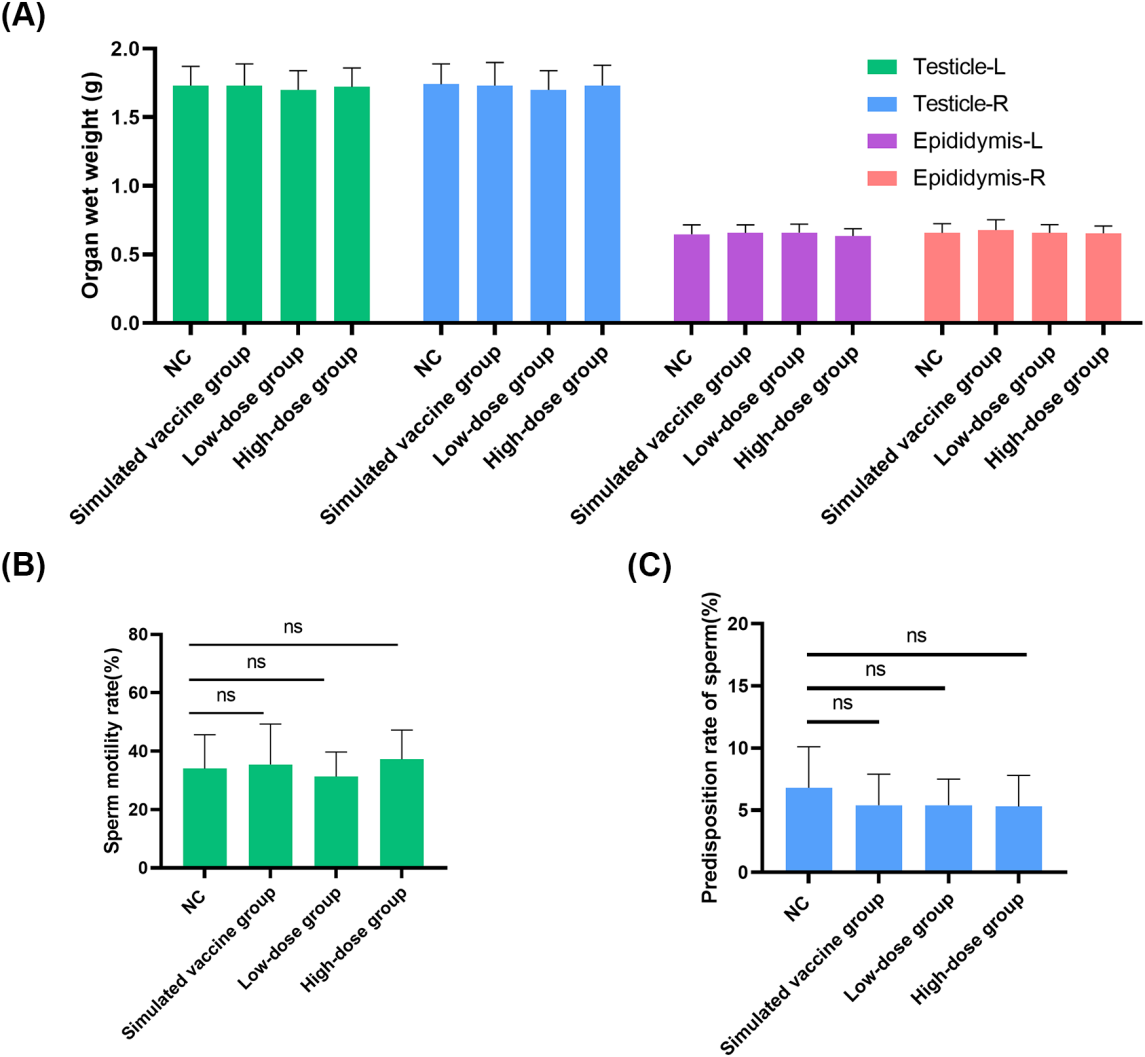
Effects of repeated intramuscular administration on male fertility in rats. The negative control group received 0.9% sodium chloride injection at 0.5 mL/rat, the mock vaccine control group received formulation buffer at 0.5 mL/rat, and the low- and high-dose groups received the quadrivalent influenza virus subunit vaccine (MDCK cell-derived) at 0.25 mL/rat and 0.5 mL/rat, respectively. **(A)** Organ wet weight of Testicle and Epididymis; **(B)** Sperm motility rate; **(C)** Predisposition rate of sperm. Each data point represents the mean value of 20 rats. Statistical comparisons were performed using ANOVA followed by Tukey’s multiple comparison test; ns, no significant difference **(A)**; **(B, C)** were performed using an independent sample t-test.

Regarding fertility parameters, no statistically significant differences were found compared with the negative control group in testis and epididymis wet weights and coefficients (**Figure 6A**), epididymal sperm motility rate (**Figure 6B**), or sperm abnormality rate (**Figure 6C**).

**Figure 7:**
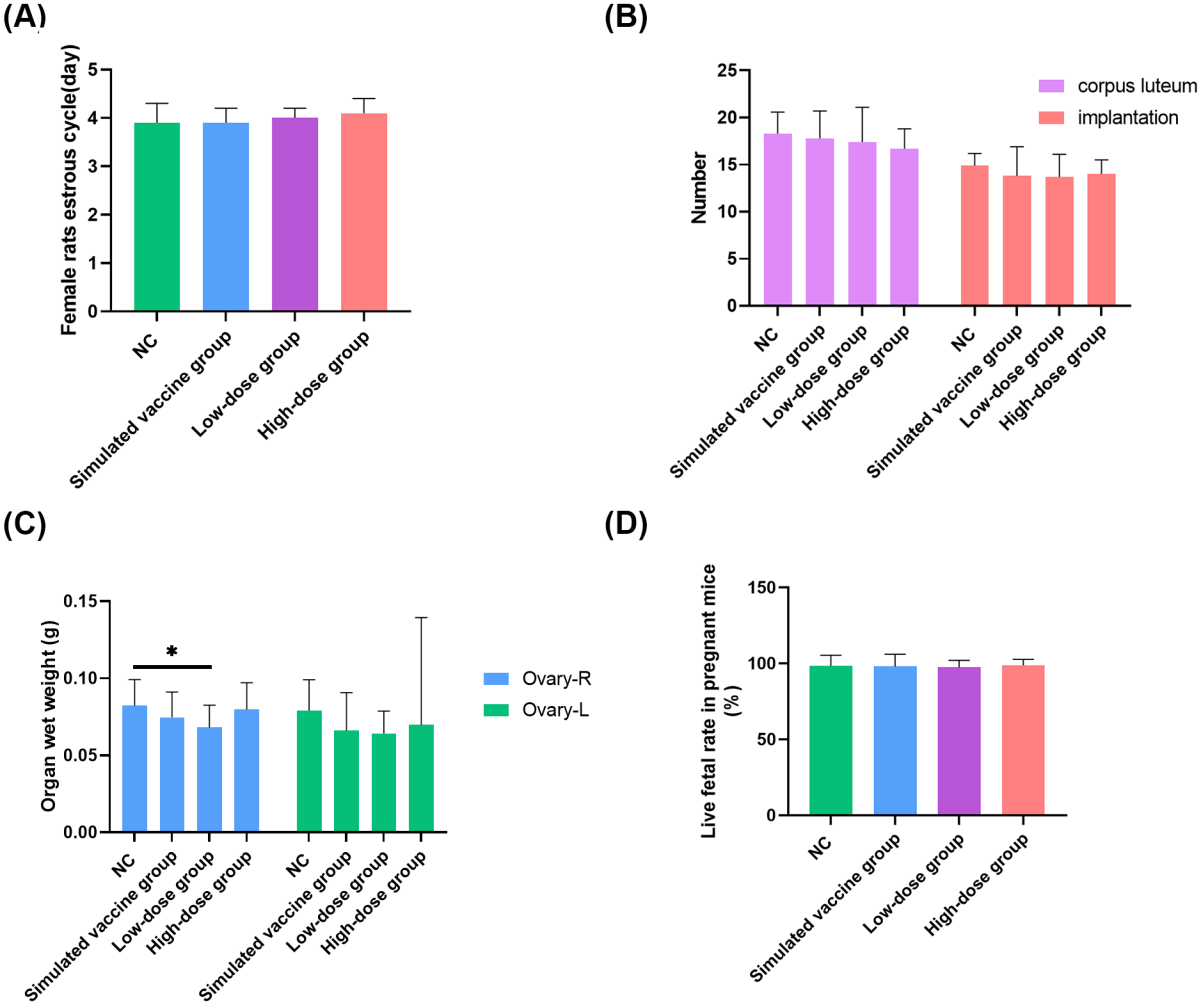
Effects of repeated intramuscular administration on female fertility and embryo-fetal development in rats. The negative control group was given 0.9% sodium chloride injection at 0.5 mL/rat, the mock vaccine control group received formulation buffer at 0.5 mL/rat, and the low- and high-dose groups were administered the quadrivalent influenza virus subunit vaccine (MDCK cell-derived) at 0.25 mL/rat and 0.5 mL/rat, respectively. **(A)** Female rats estrous cycle; **(B)** Corpus luteum number and implantation number; **(C)** Organ wet weight of Ovary; **(D)** Live fetal rate in pregnant rat. Each data point represents the mean value of 20 rats. Statistical comparisons were performed using an independent sample t-test; *p ≤ 0.05.

As shown in **Figure 7A**, the statistical results of the estrous cycle in female mice showed that the negative control group was (3.9 ± 0.4) days, the mock vaccine group was (3.9 ± 0.3) days, the low-dose group was (4.0 ± 0.2) days, and the high-dose group was (4.1 ± 0.3) days (n=20). **Figure 7B** displays that in the negative control group, the number of corpora lutea and implantation sites were (18.3 ± 2.3) and (14.9 ± 1.3), respectively; in the mock vaccine group, they were (17.8 ± 2.9) and (13.8 ± 3.1); in the low-dose group, (17.4 ± 3.7) and (13.7 ± 2.4); and in the high-dose group, (16.7 ± 2.1) and (14.0 ± 1.5).No significant effects were observed on estrous cycle (**Figure 7A**), number of corpora lutea and implantations (**Figure 7B**), or litter weight. Although the right ovary wet weight in the low-dose group (0.0680 ± 0.0146) g was statistically significantly lower than that of the negative control (0.0822 ± 0.0170) g (**Figure 7C**), the change showed no dose dependency and was therefore considered unrelated to the test article.

Regarding embryo-fetal developmental toxicity, no significant differences were observed compared to the negative control group in placental weight, fetal sex ratio, live fetus rate, dead fetus rate, or male/female fetal body weight. After re-mating following the study, successful mating occurred in all groups. The numbers of pregnant females were as follows: negative control (20), mock control (18), low-dose group (19), and high-dose group (18) (**Figure 7D**). The live fetus rate remained unaffected.

**Figure 8:**
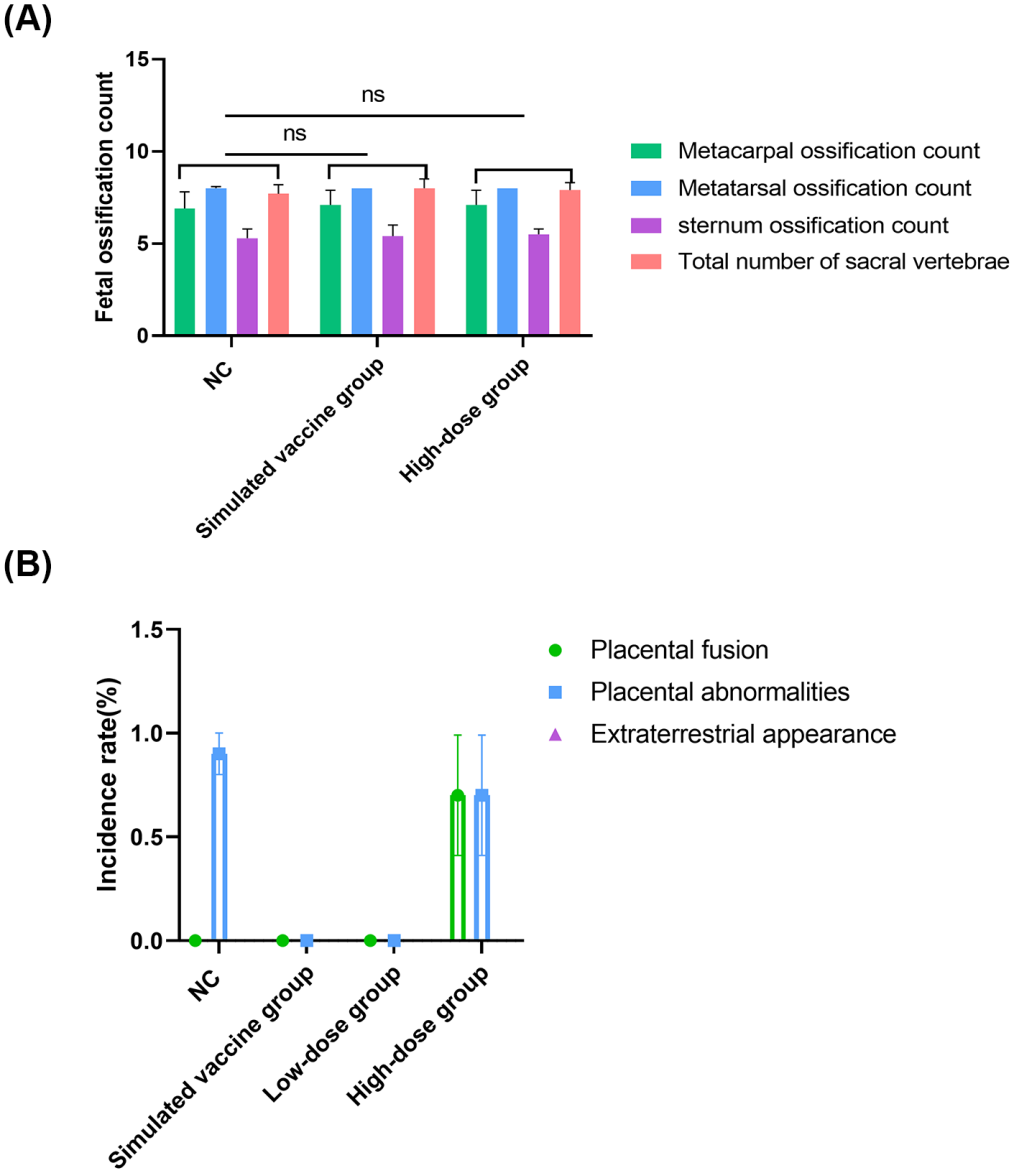
Teratogenicity assessment following repeated intramuscular administration in rats. The negative control group was administered 0.9% sodium chloride injection (0.5 mL/rat). The simulated vaccine control group received formulation buffer (0.5 mL/rat). The low- and high-dose groups were administered the quadrivalent influenza virus subunit vaccine (MDCK cell-derived) at 0.25 mL/rat and 0.5 mL/rat, respectively. **(A)** Fetal ossification count **(B)** Incidence rate of placental fusion, placental abnormalities and extraterrestrial appearance. Data are presented as the mean for each group (n=20 rats). Statistical analysis was performed using one-way ANOVA followed by Tukey’s multiple comparison test; ns denotes not significant.

In order to test the teratogenic toxicity of the vaccine and the simulated vaccine, the fetal ossification number and placental fetal appearance of each experimental group were examined and compared with the negative control group,the results showed that no statistically significant differences in the number of ossification centers for fetal metacarpals, metatarsals, sternum, and sacral vertebrae compared to the negative control (**Figure 8A**). External examination of placentas and fetuses: Negative control placental abnormality litter incidence (0.9 ± 4.1)%; simulated control (17 litters examined); Low dose (19 litters, 255 fetuses) showed no external fetal abnormalities. High dose (18 litters) showed fused placenta, placental abnormality litter incidence (0.7 ± 2.9)% (**Figure 8B**).

#### 2.1.6 Perinatal toxicity study in SD rats by intramuscular injection

No significant abnormalities in mating success rate, pregnancy rate, or pseudopregnancy rate were observed between the negative control group and the test groups. No animals exhibited premature birth, abortion, dystocia, or incomplete delivery (**Table 3**). Clinical observations, body weight, food consumption, gestation period, and the number of implantation sites in the simulated vaccine control, low-dose, and high-dose P generation dams showed no significant abnormalities.

**Table 3:** Statistical data on mating status of P generation female mice.

Observation indicator	Negative control group	Simulated vaccine control group	Low dose group	High dose group
	Incidence rate (%)	Incidence rate (%)	Incidence rate (%)	Incidence rate (%)
Successful mating	100.0	100.0	100.0	100.0
Pseudopregnancy	5.0	5.0	10.0	0.0
Pregnancy	95.0	95.0	90.0	100.0
Premature birth	0.0	0.0	0.0	0.0
Miscarriage	0.0	0.0	0.0	0.0
Difficult labor	0.0	0.0	0.0	0.0
Incomplete delivery	0.0	0.0	0.0	0.0

The results of perinatal toxicity assessment in the F1 generation showed that, compared with the negative control group, no statistically significant differences were observed in the external abnormality rate, birth survival rate, lactation survival rate, sex ratio, body weight, physical development indicators, or reflex development indicators of F1 offspring in the simulated vaccine control, low-dose, and high-dose groups (**Table 4**).

**Table 4:** F1 generation mouse survival statistics.

Observation indicators		Negative control group (0.5 mL/rat)	Simulated vaccine control group (0.5 mL/rat)	Low dose group (0.25 mL/rat)	High dose group (0.25 mL/rat)
Surface abnormality rate (%, nest)		0.3 ± 1.4	0.8 ± 3.3	0.3 ± 1.4	1.0 ± 2.5
Birth survival rate (%)	(MEAN ± SD)	98.3 ± 3.6	96.1 ± 6.6	97.8 ± 3.7	98.5 ± 3.1
Breastfeeding survival rate (%)	MEAN ± SD)	100.0 ± 0.0	100.0 ± 0.0	99.3 ± 3.0	100.0 ± 0.0
Sex ratio of offspring mice (♂:♀)		1.0(139:145)	0.9(135:145)	0.9(121:132)	1.0(146:142)

#### 2.1.7 Immunogenicity evaluation in mice

**Figure 9:**
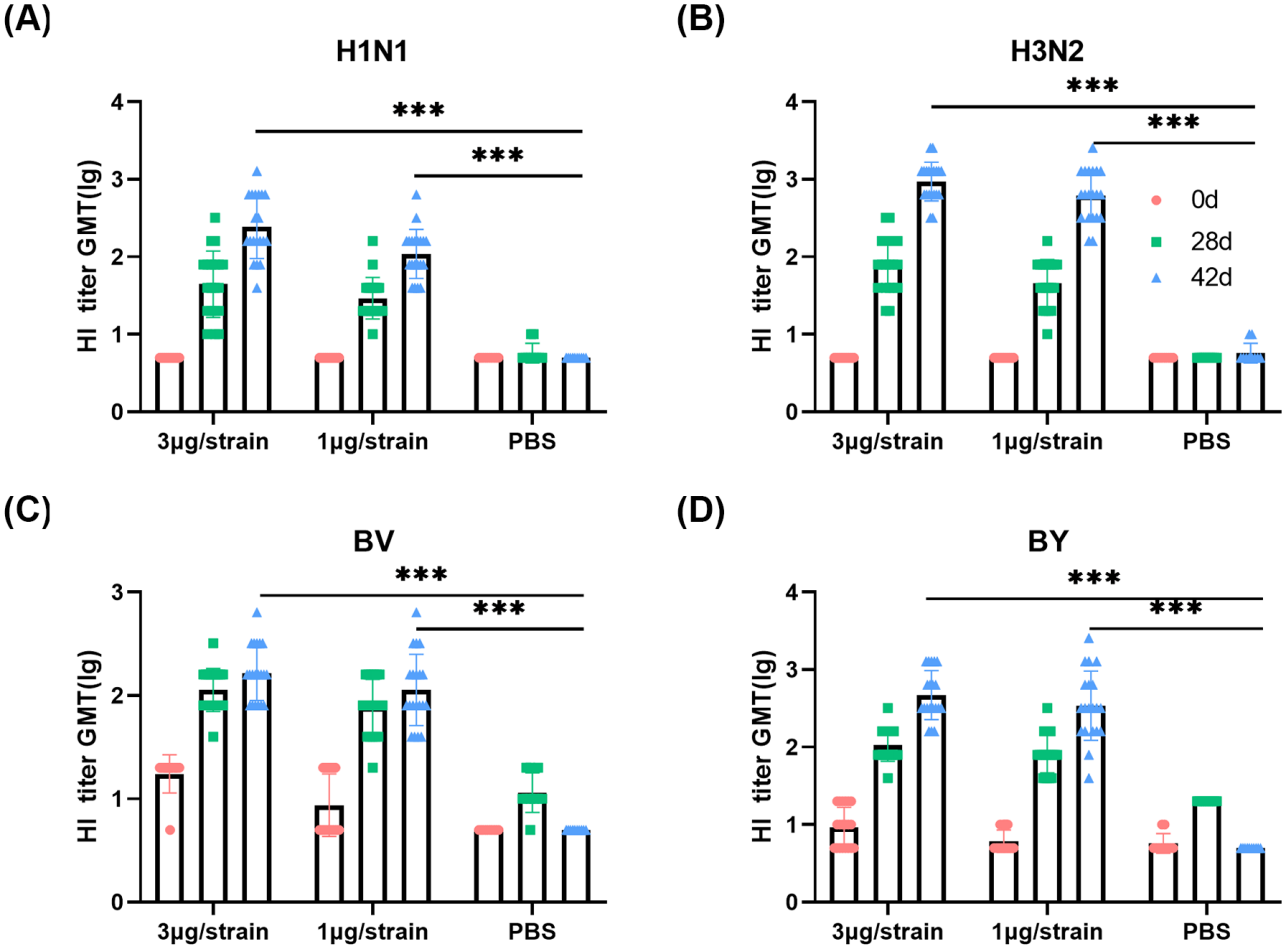
Hemagglutination inhibition (HI) antibody titers induced by quadrivalent influenza virus subunit vaccine (MDCK cell-derived).The experimental groups were injected with 3 μg HA/strain and 1 μg HA/strain, respectively, using the viral strains A/Guangdong-Maonan/SWL1536/2019, A/Kansas/14/2017 (NYMC-327), B/Maryland/15/2016 (NYMC-69A), and B/Phuket/3073/2013. The negative control group was injected with formulation buffer. **(A)** HI titer of H1N1; **(B)** HI titer of H3N2; **(C)** HI titer of BV; **(D)** HI titer of BY. Each data point represents one animal. Statistical comparisons were performed using an independent sample t-test, ***p ≤ 0.0001.

As shown in **Figure 9**, the Quadrivalent Influenza Virus Subunit Vaccine (MDCK cell-derived) stimulated the production of serum Hemagglutination Inhibition (HI) neutralizing antibodies against H1N1, H3N2, BY, and BV strains in mice 28 and 42 days after immunization. Different dose groups showed a dose-dependent response. After 42 days of immunization, the titers of HI antibodies against H1N1 virus in the 3 μg HA/strain/mouse group and 1 μg HA/strain/mouse were 49 and 22, the titers of HI antibody against H3N2 influenza virus were 187 and 124, the titers of HI antibody against BV influenza virus were 10 and 13, and the titers of HI antibody against BY influenza virus were 50 and 56, respectively. The seroconversion rate for HI antibodies against all four strains reached 100% in all test groups 42 days post-immunization.

**Figure 10:**
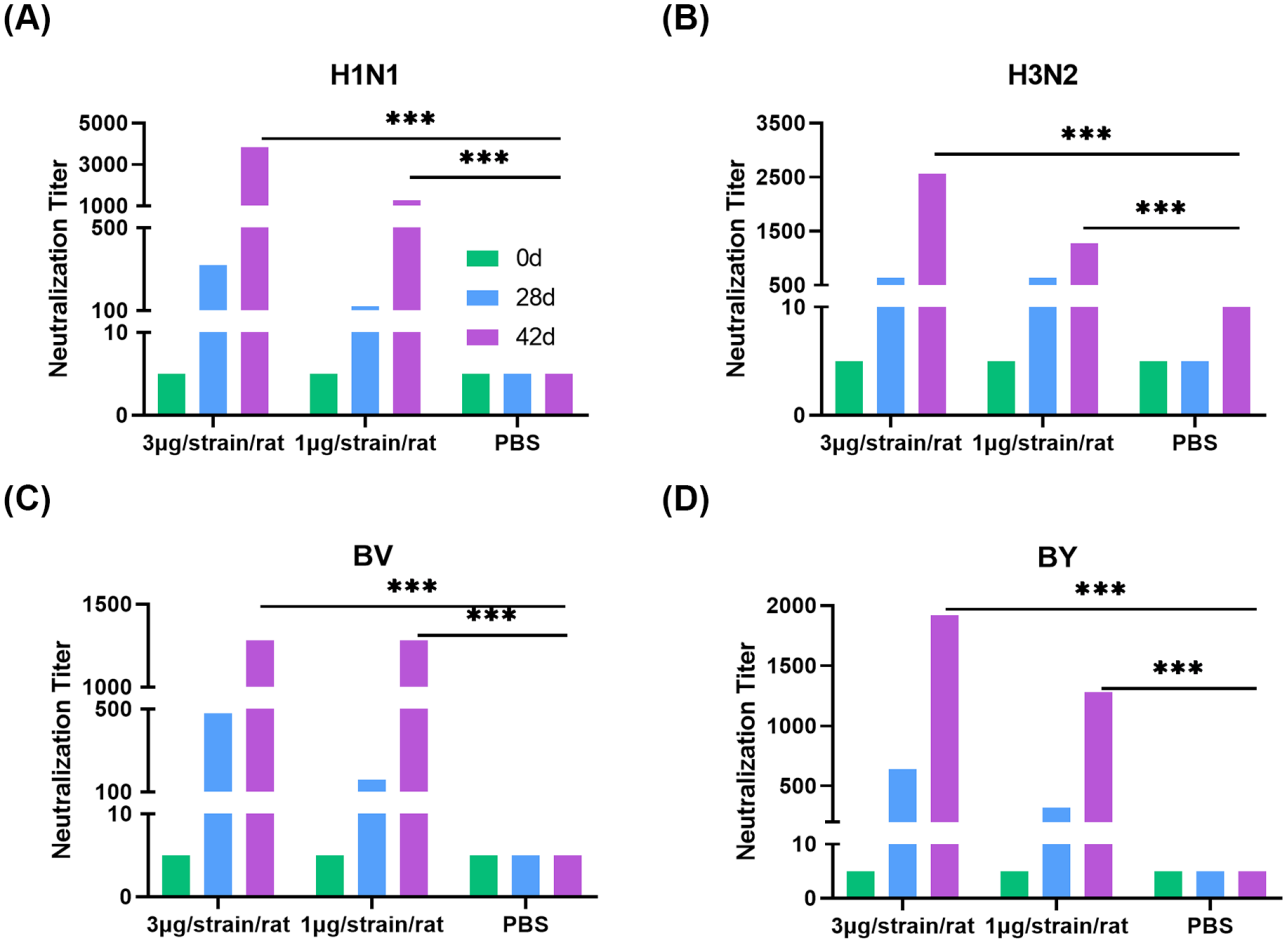
Neutralizing antibody titers induced by the quadrivalent influenza virus subunit vaccine (MDCK cell-derived). The experimental groups were injected with doses of 3 μg HA/strain/mouse and 1 μg/strain/mouse, respectively, using the viral strains A/Guangdong-Maonan/SWL1536/2019, A/Kansas/14/2017 (NYMC-327), B/Maryland/15/2016 (NYMC-69A), and B/Phuket/3073/2013. The negative control group was injected with the formulation buffer. **(A)** Neutralization titer of H1N1; **(B)** Neutralization titer of H3N2; **(C)** Neutralization titer of BV; **(D)** Neutralization titer of BY. The immunization route was intramuscular injection into the tibialis anterior muscle of the hind limb, with samples from each group pooled in a single 1.5 mL EP tube. Statistical comparisons were performed using an independent sample t-test, ***p ≤ 0.0001.

As shown in **Figure 10**, the vaccine also induced the production of microneutralization (MN) antibodies against H1N1, H3N2, BY, and BV strains in mice at 28 and 42 days post-immunization, demonstrating a dose-dependent effect. On day 42 post-immunization, the neutralizing antibody titers (1:X) against the H1N1 influenza virus strain were 3840 and 1280 in the groups immunized with 3 μg HA/strain/mouse and 1 μg HA/strain/mouse, respectively. The corresponding titers against the H3N2 strain were 2560 and 1280; against the BV strain, 1280 and 1280; and against the BY strain, 1920 and 1280.

## 3 Methods

### 3.1 Vaccine preparation

Virus strains A/Guangdong-Maonan/SWL1536/2019, A/Kansas/14/2017 (NYMC-327), B/Maryland/15/2016 (NYMC-69A), and B/Phuket/3073/2013 were used to infect suspension MDCK(ATCC, NBL-2) cells. MDCK cells were domestically into high-density suspension culture cells and cultured in MS01A (T) medium. The virus was inoculated at an MOI of 10^-^³ to 10^-5^ and cultured at 34 °C for 3 days. The harvested virus fluid was purified using affinity chromatography, and the target protein was collected at 280 nm. After virus inactivation and disruption, the sample was further purified by density gradient centrifugation, 30000rpm 3h. The gradient centrifugation fractions were collected and subjected to ion chromatography for additional purification. Following concentration, the product was sterile-filtered through 0.45 μm and 0.22 μm filters to obtain monovalent bulk substances. Based on the hemagglutinin content of each monovalent bulk, the semi-finished product was formulated with each influenza virus type at 30 μg/mL hemagglutinin. The finished product was obtained after filling and packaging (registration number: ChiCTR2400083316).

### 3.2 Single-dose toxicity study in SD rats by intramuscular injection

The “General principles for technical review of preclinical safety evaluation of preventive biological products” (21) recommend that long-term animal studies and toxicological tests for vaccines should be conducted in relevant species or strains of animals. Therefore, acute toxicity studies, repeated dose toxicity studies, and reproductive toxicity studies were conducted using the commonly employed rodent species, Sprague-Dawley (SD) rats, for toxicological assessment. The study used 40 SD rats, half male and half female. Intramuscular injection was administered at a volume of 1.0 mL/rat. The negative control group received 0.9% sodium chloride injection, and the test article group received the Quadrivalent Influenza Virus Subunit Vaccine (MDCK cell-derived). Animals were observed at fixed intervals post-dosing, with local reactions observed daily for 14 consecutive days. Body weight was measured twice weekly before and after dosing. All animals underwent gross necropsy, and organs with gross lesions were subjected to histopathological examination.

### 3.3 Repeated-dose toxicity study in SD rats via intramuscular injection

SPF-grade SD rats were used and divided into four main experimental groups: negative control group, simulated vaccine control group, low-dose group, and high-dose group, with 40 animals per group (half male, half female). Intramuscular injection was used for administration. The negative control group received 0.9% sodium chloride injection, and the simulated vaccine control group received the formulation buffer. The administration volume was 0.5 mL per animal. The low- and high-dose groups received the quadrivalent influenza virus subunit vaccine (MDCK cell-derived), with administration volumes of 0.25 mL and 0.5 mL per animal, respectively. Administration was performed on D0, D14, and D28 (D0 represents the first administration day), for a total of three administrations. There was a 28-day recovery period after the last administration. During the study, clinical signs were observed daily, and body weight and food consumption were measured weekly. Hematology, coagulation, and serum biochemistry tests were performed on D3. Ophthalmic and urinalysis examinations were planned on R2 and R27 (R2 represents 2 days after the last administration), followed by necropsy. On R3 and R28, hematology, serum biochemistry, coagulation tests, CD4^+^/CD8a^+^ T-cell counts, cytokine levels, and anti-nuclear antibody detection were performed. After necropsy, bone marrow smears were prepared, organ wet weights were measured, and tissue samples were collected for histopathological examination.

As for the detection of CD4^+^ and CD8^+^ T-Cells, after an overnight fast, the animals were anesthetized by subcutaneous injection of tiletamine hydrochloride and zolazepam hydrochloride (30 mg/kg; 50 mg/mL, 0.6 mL/kg) in the dorsal cervical region. Approximately 1.5 mL of blood was then collected from the abdominal vena cava and placed into heparinized anticoagulant tubes. The tubes were thoroughly shaken to mix and anticoagulate. About 30 µL of whole blood was used to prepare samples for flow cytometry analysis, while the remaining blood was used for cytokine measurement, add 30 µL of pre-mixed fluorescence-labeled monoclonal antibodies against CD3, CD4, and CD8a, and incubate for 15–20 minutes at room temperature in the dark. Then add 2 mL of red blood cell lysis buffer and incubate for 10–15 minutes at room temperature in the dark. Centrifuge at 300–350 x*g* for 5–10 minutes, discard the supernatant, and wash the cells once with staining buffer. Resuspend the cell pellet in PBS containing 1% paraformaldehyde or staining buffer, and analyze on a flow cytometer. For data analysis, first the lymphocyte population was gated on an FSC/SSC scatter plot, doublets were excluded, and then CD3^+^ T-cell were obtained by gating on CD3/SSC (22).The proportions of CD3^+^CD4^+^ and CD3^+^CD8a^+^ cells within the lymphocyte population were analyzed using GuavaSoft software, and the CD4^+^/CD8^+^ ratio was subsequently calculated.

Cytokine measurement was performed using the whole blood aliquot prepared for CD4^+^/CD8a^+^ T−cell detection, which was centrifuged at 3000 rpm for 10 minutes, the concentrations of cytokines IFN−γ, IL−2, and IL−4 were measured using a Cytometric Bead Array (CBA) assay. Briefly, samples and a series of standards were incubated with capture antibody−coated beads to form bead−cytokine complexes. After incubation, PE−conjugated detection antibodies were added to generate sandwich immunocomplexes. Following washes, the bead mixtures were analyzed on a Guava easyCyte flow cytometer. Cytokine concentrations were determined based on the corresponding standard curves (23). IL-6 levels were measured using a double-antibody sandwich ELISA kit. Serum was separated by centrifugation at 2,000-3,000 rpm for 20 minutes after clot formation. Standards or serum samples (100 μL) were added to pre-coated wells, incubated for 2 hours at 37 °C, followed by sequential incubations with biotinylated detection antibody (1 hour, 37 °C) and horseradish peroxidase HRP-conjugate (30 minutes, 37 °C). After washing, tetramethylbenzidine (TMB) substrate was added for 15–20 minutes at room temperature, and the reaction was stopped. Optical density (OD) values were read at 450 nm, and IL-6 concentrations were calculated from a standard curve.

The antinuclear antibody test uses serum separated by serum biochemistry and is performed by indirect immunofluorescence assay under a fluorescent biological microscope (with BV/UV excitation light) at 200× or 400× magnification. Positive results are indicated by apple-green fluorescence appearing in the nucleus, cytoplasm, or nucleolus.

### 3.4 Muscle irritation test in rabbits

The “Technical Guidelines for Drug Irritation, Allergy, and Hemolysis Studies” (24) recommend that irritation tests should be conducted in animal species that exhibit responses similar to those of human skin or mucous membranes, such as rabbits or miniature pigs. Therefore, New Zealand rabbits were selected as the test animals. Four healthy Japanese white rabbits were used, employing a self-control method comparing left and right sides. The finished product of the quadrivalent influenza virus subunit vaccine (MDCK cell-derived) and an equal volume of 0.9% sodium chloride injection were administered via intramuscular injection into the left and right quadriceps muscles, respectively, with an administration volume of 0.5 mL per side per animal, as a single dose. Half of the animals were euthanized 48 hours after administration, and the other half after an additional 14-day recovery period. The quadriceps muscles were dissected, longitudinally sectioned, and visually observed for irritation reactions at the injection site, with corresponding scores assigned. Tissues were fixed in 10% neutral buffered formalin and trimmed, processed with graded ethanol dehydration, embedded in paraffin, and sectioned at approximately 3 μm thickness using a rotary microtome. Sections were stained with hematoxylin and eosin (H&E) and examined under light microscopy.

### 3.5 Active systemic anaphylaxis test in guinea pigs

The “General Principles for Technical Evaluation of Non-clinical Safety Assessment of Preventive Biological Products” (21) recommend that vaccines complete conventional guinea pig systemic active test prior to clinical trial. Therefore, guinea pigs were selected as the test animals for the allergy study. Thirty-two healthy guinea pigs (half male, half female) were assigned to four groups: a negative control group, a positive control group, a low-dose test article group, and a high-dose test article group. During the sensitization phase, the negative control group received 0.5 mL of 0.9% sodium chloride injection; the positive control group was administered 0.25 mL of 0.1% high-purity ovalbumin; and the low- and high-dose test groups were given 0.25 mL and 0.5 mL, respectively, of the finished quadrivalent influenza virus subunit vaccine (MDCK cell-derived).

All treatments were delivered via intramuscular injection every other day for a total of three administrations. For challenge, half of the animals from each group (2 females/2 males) were selected at 14 and 21 days after the last sensitization and injected intravenously in the footpad with twice the volume of the respective sensitizing concentration. All reactions occurring immediately after challenge were recorded until resolution; in the absence of symptoms, observation continued for 30 minutes. Anaphylactic responses to the test article were evaluated based on established anaphylactic reaction scoring criteria.

### 3.6 Fertility and early embryonic development toxicity and embryo-fetal development toxicity in SD rats via intramuscular injection

The “General Principles for Technical Evaluation of Non-clinical Safety Assessment of Preventive Biological Products” (21) recommend that long-term animal studies and toxicological tests for vaccines should be conducted in relevant species or strains of animals. Therefore, for reproductive toxicity studies, as well as acute toxicity studies and repeated dose toxicity studies, the routinely used rodent species, Sprague-Dawley (SD) rats, were selected for toxicological assessment. Sexually mature SD rats, comprising 81 females and 80 males, were randomly assigned to four groups by sex and body weight using TOXSTAT2006 software: a negative control group (0.9% sodium chloride injection, 0.5 mL/rat), a simulated vaccine control group (formulation buffer, 0.5 mL/rat), a low-dose group (quadrivalent influenza virus subunit vaccine, 0.25 mL/rat), and a high-dose group (quadrivalent influenza virus subunit vaccine, 0.5 mL/rat). All treatments were administered via intramuscular injection. Male rats received three doses at 5, 3, and 1 week before mating, while female rats were dosed at 3 and 1 week before mating and on gestation day 6. For mating, female and male rats were paired 1:1 within the same group. The study included observation of clinical signs, body weight and food consumption measurements, vaginal smears, necropsy, blood collection, and antibody detection.

### 3.7 Perinatal toxicity study in SD rats via intramuscular injection

Sexually mature Sprague-Dawley rats, comprising 80 females and 40 males, were utilized in the study. Eighty female rats were randomly divided into four groups by body weight using TOXSTAT2006 software: a negative control group, a simulated vaccine control group, a low-dose group, and a high-dose group. The male rats were exclusively used for mating and were not assigned to experimental groups. Female rats in each group received four intramuscular administrations: at 3 weeks and 1 week before mating, on gestation day 6, and on postpartum day 7. The negative control group received 0.9% sodium chloride injection (0.5 mL/rat), the simulated vaccine control group received formulation buffer (0.5 mL/rat), and the low- and high-dose groups received the quadrivalent influenza virus subunit vaccine (MDCK cell-derived) at 0.25 mL/rat and 0.5 mL/rat, respectively. For mating, male and female rats were housed together in a 1:1 ratio. The following morning, vaginal plugs were checked; in females lacking a visible plug, vaginal smears were examined for the presence of sperm. Detection of a vaginal plug or sperm was considered evidence of successful mating, and the corresponding day was designated as gestation day 0. The study parameters included observation of clinical signs, measurement of body weight and food consumption, assessment of parturition conditions, examination of F1 offspring, necropsy, blood collection, and antibody detection.

### 3.8 Immunogenicity evaluation in mice

The “Technical Guidelines for Non-clinical Studies of Preventive Vaccines” (25)recommends that the immunogenicity of vaccines should be directly evaluated using animal models. Therefore, referring to studies on similar influenza vaccines, Kunming mice were selected as the species for the primary pharmacodynamic immunogenicity test. Fifty SPF-grade healthy female Kunming mice were used. The mice were randomly divided into three groups: two experimental groups (20 mice each) and one negative control group (10 mice). The two experimental groups received injections at doses of 3 µg HA/strain/mouse and 1 µg HA/strain/mouse, respectively. The negative control group received the formulation buffer. The immunization route was intramuscular injection into the hind limb tibialis anterior muscle. All groups were housed under identical conditions. Immunizations were performed on day 0 and day 28. Blood was collected on days 0, 28, and 42. Serum was separated, and HI and MN antibody titers were measured as described (23).

Serum antibody titers were determined by the standard HI assay using a 96-well V-bottom microtiter plate. Briefly, serum samples were heat-inactivated at 56°C for 30 min and treated with receptor-destroying enzyme (RDE) to remove nonspecific inhibitors. Viral antigen was titrated to determine one hemagglutinating unit (1 HAU), and a working solution containing 4 HAU was prepared and back-titrated for verification. Serial two-fold dilutions of treated serum (starting from 1:10 or 1:20) were incubated with 4 HAU of antigen at room temperature for 20–30 min. Then, a 1% chicken red blood cell suspension was added to each well. After incubation at room temperature for 30–40 min, the HI titer was recorded as the reciprocal of the highest serum dilution that completely inhibited hemagglutination. Negative and positive serum controls as well as red blood cell controls were included in each test.

Serum neutralizing antibody titers were determined by a standard microneutralization assay using the constant virus–diluting serum method. Briefly, heat-inactivated (56 °C, 30 min) serum samples were subjected to two-fold serial dilutions (starting from 1:8 or 1:10) in a 96-well cell culture plate. An equal volume of pre-titrated virus solution (100 TCID_50_) was added to each well containing diluted serum. After incubation at 37 °C with 5% CO_2_ for 1–2 h for neutralization, the serum-virus mixtures were transferred onto monolayers of susceptible cells. The plates were then incubated at 37 °C with 5% CO_2_ for 3–7 days. Cytopathic effect (CPE) was monitored daily under an inverted microscope. The assay was considered valid only when the virus control wells showed complete CPE and the cell control wells remained normal. The 50% neutralization titer was calculated using the Reed-Muench method and defined as the reciprocal of the highest serum dilution that protected 50% of the cells from CPE.

### 3.9 Animals

A total of 520 6-8-week-old specific pathogen-free (SPF) rats (half male and half female) were purchased from Beijing Vitong Lihua Laboratory Animal Technology Co., Ltd. (Beijing, China) for a series of non-clinical safety pharmacology studies. The studies included: a single-dose toxicity study (n=40; IACUC No. XB-IACUC-2022-0941), a repeated-dose toxicity study (n=190; IACUC No. XB-IACUC-2022-0942), a combined fertility and early embryonic development, and embryo-fetal development toxicity study (n=120; IACUC No. XB-IACUC-2022-0944), and a perinatal toxicity study (n=170; IACUC No. XB-IACUC-2022-0943). All studies were conducted at and approved by the Institutional Animal Care and Use Committee (IACUC) of the Shandong Xinbo Drug Safety Evaluation and Research Center.

6-8-week-old SPF healthy Kunming mice, female (n=50), were provided by the Laboratory Animal Center of Changchun Institute of Biological Products Co., Ltd. (IACUC Issue No.: CCIBP-IACUC-2022-043834)(Changchun, China) and used for an immunogenicity study.

Two male and two female conventional-grade Japanese White rabbits were purchased from Qingdao Konde Aibo Biotechnology Co., Ltd. (Qingdao, China), and thirty-two SPF-grade guinea pigs (half male and half female) were also obtained from the same supplier. All animal experiments were approved by the Institutional Animal Care and Use Committee (Approval No. XB-IACUC-2022–0976 for rabbits and No. XB-IACUC-2022–0975 for guinea pigs).

All animal experiments were approved by the Animal Ethics Committee of the Shandong Xinbo Drug Safety Evaluation and Research Center and Changchun Institute of Biological Products Co., Ltd. All procedures were performed in accordance with the National Institutes of Health (NIH) Guide for the Care and Use of Laboratory Animals. Every effort was made to minimize animal suffering. Before introducing the animals, do not pre-fill the chamber with CO_2_. After placing the animals inside, introduce CO_2_ into the chamber at a rate that replaces 10% - 30% of the chamber volume per minute. Once the animals are immobile, have ceased breathing, and their pupils are dilated, turn off the CO_2_. Then observe for an additional 2–3 minutes to confirm death. Finally, cervical dislocation (applicable to mice weighing <200g) or bilateral pneumothorax must be immediately performed as an irreversible means of euthanasia to ensure the animal is completely dead and cannot recover. The above operation complies with the Chinese national standard GB/T 39760-2021.

## 4 Discussion

Traditional egg-based production technology for seasonal influenza vaccines has several drawbacks, including lack of flexibility, dependence on egg supply, risk of contamination, and egg-adapted viral mutations. The vaccine developed in this study is not an immunomodulator, so immunotoxicity studies were not conducted. It does not act on the central nervous system, shows no potential for dependence in humans, and displayed no dependency potential in completed non-clinical studies; therefore, dependency studies were not conducted. This product has no metabolites, so metabolite studies were not performed. Consequently, the safety evaluation conducted in this study included single-dose toxicity, repeated-dose toxicity, reproductive toxicity, anaphylaxis, and irritation studies. Immunogenicity results demonstrated that the product effectively induces immune responses against the four types of influenza viruses.

A single-dose toxicity study in SD rats administered the quadrivalent influenza virus subunit vaccine (MDCK cell-derived) intramuscularly at two doses showed no abnormalities in clinical signs or local reactions, normal body weight gain, and no significant gross pathological changes upon necropsy of surviving animals at the end of the recovery period. A repeated-dose toxicity study in SD rats administered the vaccine intramuscularly at 0.5 and 1 dose showed no significant abnormalities in hematology, serum biochemistry, coagulation, CD4^+^/CD8a^+^ T-cell, cytokines, or anti-nuclear antibodies after three administrations. No significant histopathological changes were observed in any tissues or organs. Single intramuscular administration of the quadrivalent influenza virus subunit vaccine (MDCK cell-derived) in Japanese white rabbits showed no drug-related irritation. The systemic sensitization evaluation in guinea pigs administered the vaccine yielded negative results. The fertility and early embryonic development toxicity and embryo-fetal development toxicity study in SD rats administered 0.25 mL and 0.5 mL of the vaccine intramuscularly indicated no significant effects on body weight, food consumption, fertility, or early embryonic and fetal development after three administrations. The perinatal toxicity study in SD rats administered 0.5 or 1 dose of the vaccine intramuscularly indicated no abnormalities in food consumption, body weight, or gestation period parameters in the P-generation dams, and no abnormalities in survival rate at birth, survival rate during lactation, sex ratio, external abnormality rate, body weight, physical development, or reflex development indicators in the F1 offspring after four administrations, indicating no perinatal toxicity. These conclusions collectively confirm that the quadrivalent influenza virus subunit vaccine (MDCK cell-derived) is safe and effective compared with the research results of Ye H et al (26). Following clinical evaluation, it could serve as a candidate vaccine for the prevention and control of influenza pandemics.

The effectiveness of the vaccine can be verified by the hemagglutination inhibition experiment and serum neutralization test after animal immunization, and the WHO stipulates that the HI antibody after influenza vaccine immunization should be ≥ 1:40 or more than 4 times to be judged positive (27), and the quadrivalent influenza virus subunit vaccine was developed based on the MDCK suspension cell platform, and the positive conversion rate of anti-H1N1, H3N2, BY and BV influenza virus HI antibodies in the serum of mice in each experimental group after 42 days of immunization was 100%, and the anti-influenza virus-specific HI antibody titers were 100% (GMT) increased by more than 22 times compared with the average before immunization. After 28 days and 42 days of immunization of mice, mice were stimulated to produce neutralizing antibodies against H1N1, H3N2, BY and BV virus MN, and were dose-dependent. Compared to the study by Ye H et al., our 28-day GMTs of HI antibodies against influenza strains among different groups of BALB/c mice were lower than those reported by Ye H et al, but the 42-day GMTs were higher (26).To overcome these limitations, the EU and FDA have approved MDCK cell-based influenza subunit vaccines. Extensive clinical data indicate that cell-based influenza vaccines are comparable to egg-based influenza vaccines in terms of immunogenicity, safety, and tolerability (13, 28–30). This study evaluated the immunogenicity and safety of a quadrivalent influenza virus subunit vaccine (MDCK cell-derived). Immunogenicity, a key indicator of vaccine efficacy, refers to the strength and duration of the immune response, including antibody production, elicited by the vaccine in the body. Factors affecting immunogenicity include host-related factors and vaccine-related factors. The immunogenicity of this product was evaluated by measuring HI and MN antibody titers in mouse serum.

The “WHO guidelines on non-clinical evaluation of vaccines.2005” (31)state that developmental toxicity studies are usually not required for vaccines intended for childhood immunization. However, if the target population includes pregnant women and women of childbearing potential, developmental toxicity studies should be considered unless the manufacturer provides scientifically and clinically justified arguments indicating that such studies are unnecessary. Genetic toxicity studies are typically not required for the final vaccine formulation unless it contains special components, such as novel adjuvants and additives. Carcinogenicity studies are not required for vaccine antigens unless they contain special components, such as novel adjuvants and additives. The Technical Review General Principles for Preclinical Safety Evaluation of Preventive Biological Products (32) indicates that genetic toxicity and carcinogenicity studies are usually not required for vaccines. Since no adjuvants were added to this product and all excipients are conventional, related studies on genetic toxicity and carcinogenicity were not conducted. In this study, the hemagglutination inhibition antibodies and positive conversion rate of mice immunized with quadrivalent influenza virus subunit vaccine (MDCK cell-derived) reached a qualified level, and the immunogenicity was stronger than that of quadrivalent influenza virus lysis vaccine (33).This quadrivalent influenza virus subunit vaccine (MDCK cell-derived) represents a more modern and precise production process, and its core value is to provide safe and effective antibody protection that is highly matched to the circulating strain, and hence can be an important tool for the prevention of seasonal influenza.

However, the immunogen form of its purified protein determines its natural shortcomings in stimulating cellular immunity that provides cross-protection and rapid infection control (34).This is not a unique flaw, but a common problem of the classical subunit vaccine platform. The future upgrade path is clear: using the advantages of its high-quality antigen production platform to combine strongly with new adjuvants or antigenic components that can effectively stimulate cellular immunity, it is expected to develop the next generation of influenza vaccines that can not only provide precise prevention but also achieve broad protection.

### 4.1 Statistical analysis

Animal immunogenicity data was analyzed using GraphPad PRISM software. GMTs of the immune responses for vaccine groups and strains were calculated and are displayed in each bar chart. An analysis of variance (ANOVA) was conducted to compare immune responses among NC, Simulated vaccine, Low-dose group, and High-dose group. Data are expressed as the mean ± SD. All comparisons of the four groups were performed and Tukey’s test was applied to adjust for multiple comparisons. A p-value less than 0.05 was considered statistically significant and is marked with asterisk(s) in the bar charts. Statistical analysis of body weight, body weight change, food consumption, and wet weight of organs were conducted. Descriptive statistics were generated for each parameter and group at each scheduled sampling time or each time interval. A p-value of less than 0.05 was considered statistically significant and is marked with asterisk(s) in the bar charts.

## Data Availability

The datasets presented in this study can be found in online repositories. The names of the repository/repositories and accession number(s) can be found in the article/supplementary material.
